# Minimal clinically important difference of commonly used patient-reported outcome measures in total knee arthroplasty: review of terminologies, methods and proposed values

**DOI:** 10.1186/s43019-020-00038-3

**Published:** 2020-04-09

**Authors:** Siddhartha Maredupaka, Prashant Meshram, Manish Chatte, Woo Hyun Kim, Tae Kyun Kim

**Affiliations:** TK Orthopedic Surgery, 55 Dongpangyo-ro, Bundang-gu, Seongnam-si, Gyeonggi-do 13535 Republic of Korea

**Keywords:** Knee osteoarthritis, Total knee arthroplasty, Minimal clinically important difference, MCID, Minimal clinical difference

## Abstract

**Purpose:**

The aim of this article was to highlight various terminologies and methods of calculation of minimal clinically important difference (MCID) and summarize MCID values of frequently used patient-reported outcome measures (PROMs) evaluating total knee arthroplasty (TKA).

**Materials and methods:**

PubMed and EMBASE databases were searched through May 2019. Of 71 articles identified, 18 articles matched and underwent a comprehensive analysis for terminologies used to indicate clinical significance, method of calculation, and reported MCID values.

**Results:**

MCID was the most common terminology (67% studies) and anchor-based methods were most commonly employed (67% studies) to calculate it. The analytical methods used to calculate and the estimated values of MCID for clinical use are highly variable. MCID values reported for WOMAC scores are 20.5 to 36.0, 17.6 to 33.0 and 12.9 to 25.0 for pain, function and stiffness sub-scales, respectively, and 4.7 to 10.0 for OKS.

**Conclusion:**

There was lack of standardization in the methodology employed to calculate MCID in the available studies. MCID values reported in this review could be used for patients undergoing TKA, although caution is advised in their interpretation and application.

## Introduction

Patient-reported outcome measures (PROMs) are frequently incorporated in clinical research as key outcome variables for the evaluation of the treatment effects after total knee arthroplasty (TKA) [1]. While providing patient’s inputs using self-completed scientific questionnaires, PROMs help in a better understanding of the patient’s perspective and improve physician-patient communication [2]. Consequently, numerous PROMs have been validated, including generic PROMs such as the 36-item Short Form survey (SF-36), 12-item Short Form survey (SF-12) and disease- or joint-specific PROMs such as the Western Ontario and McMaster Universities Arthritis Index (WOMAC), Oxford Knee Score (OKS), and Knee Society Score (KSS) [3–7]. However, an accurate and meaningful interpretation of the PROMs is challenging, as the traditionally reported statistically significant differences do not necessarily imply clinically meaningful change. Furthermore, statistical significance, which is centered on testing a null hypothesis using statistically determined probability “*p* value” does not provide adequate insights to make better treatment decisions [8]. Hence, interpreting the clinical research in terms of the clinical rather than statistical significance has attracted researchers in order to facilitate an evidence-based approach to clinical decision-making.

Introduced as a benchmark of reporting clinical significance, the concept of minimal clinically important difference (MCID) has emerged as an important psychometric property for interpreting changes in the PROM scores from the patient’s perspective [9]. MCID has garnered lot of attention from clinicians and researchers with its potentially wide applications in research, practice, and policy-making. In its group level application in clinical research, MCID is used as a decision threshold to test the effectiveness of a promising new treatment against the current best practice [8]. Additionally, at an individual level, it assists in preoperative discussions regarding patient expectations and helps in making balanced treatment decisions in clinical practice [10]. Moreover, with the potential to interpret the usefulness of different forms of interventions, MCID helps in the formulation of health policies by subsidizing treatments with better patient-reported improvements [11]. Hence, orthopedic surgeons should be familiar with the concept and critical issues related to MCID.

The understanding and utility of MCID in the context of TKA is challenged by the multiple similar terminologies, analytic methods used for calculation, and consequentially, wide variability in the calculated MCID values. Firstly, it has been noted that multiple terminologies are currently utilized to indicate clinically significant changes. Although certain terms like minimal clinically important change (MCIC) are interchangeably used with MCID, distinctions between other terminologies such as minimal important difference (MID), minimal important change (MIC), clinically important difference (CID), and minimum detectable change (MDC) need to be understood [11]. Secondly, multiple methods are currently available to calculate MCID which may have led to varied MCID values and confusion in choosing the appropriate method and the calculated MCID value [10]. Thirdly, there is considerable variability in MCID calculated across different studies for each of the PROMs [12–15]. It is undetermined whether this variability is because of diverse methodologies of calculation or different clinical contexts in each of the studies (such as heterogeneous demographic characteristics, disease severity, baseline PROM scores, and time-points of analysis). These critical issues need to be thoroughly reviewed before considering the application of MCID in clinical and research contexts pertinent to TKA.

The purpose of this article is to help clinicians and researchers understand the concept and critical issues related to MCID by highlighting various terminologies, methodologies used for calculation, and reported MCID values of commonly used PROMs evaluating outcomes of TKA.

## Materials and methods

A comprehensive search of the PubMed and EMBASE databases was conducted from their years of inception through May 2019, keeping the purpose of article in mind. The search was conducted by two independent reviewers (SM and MC) and limited to peer-reviewed articles in English language only. The medical subject headings (MeSH) or the keywords used for search included “minimal clinically important difference” or “MCID,” “minimal important change,” or “MIC,” “minimal important difference” or “MID”, “‘clinically important difference” or “CID,” “minimal clinically important change” or “MCIC,” and “total knee arthroplasty.” After removal of the duplicates (*n* = 361), 1520 articles matched our search criteria using the aforementioned items, including four articles obtained from manual searching from the references of the core articles. A preliminary screening of titles was performed and 992 articles were excluded as they were considered irrelevant to the current review. Five hundred and twenty-eight abstracts were thus obtained and analyzed, of which 457 articles were excluded based on a-priori established inclusion and exclusion criteria. The full text of 71 articles that passed preliminary screening were retrieved and assessed for eligibility.

**Fig. 1:**
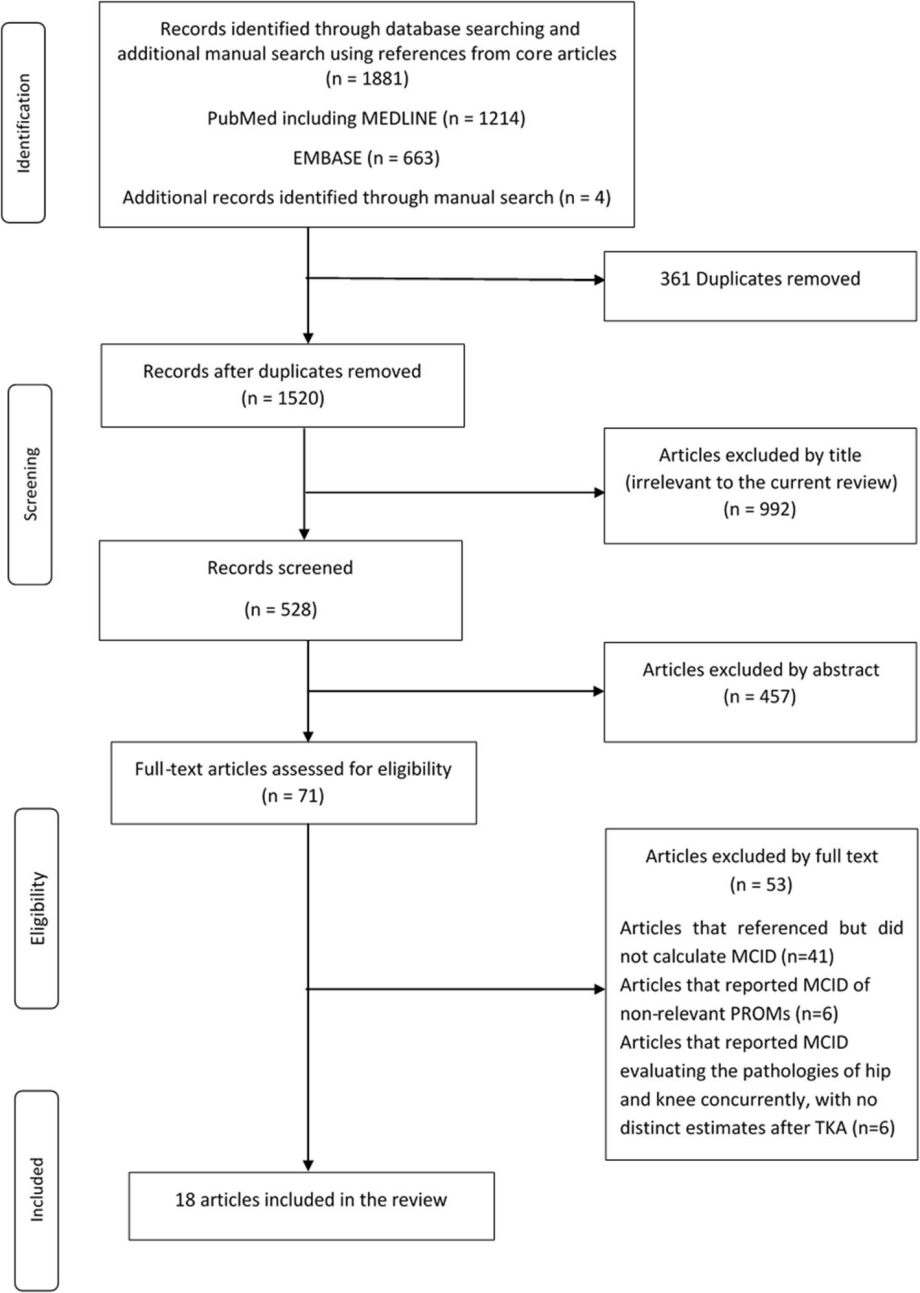
Flow chart of the article selection process

The articles were considered eligible if they reported MCID for one of the six commonly utilized PROMs evaluating outcomes of primary TKA in osteoarthritis (OA) knee which were the WOMAC, OKS, 1989 - original KSS, 2011- new KSS, SF-36 and SF-12. The articles were excluded if (1) brief reference of MCID was available but details of its calculation were missing (*n* = 41), (2) reported MCID of non-relevant PROMs (*n* = 6), (3) MCIDs were calculated for outcomes of hip and knee arthroplasty together with no distinct estimates for TKA (*n* = 6) (Fig. 1). Finally, 18 studies were considered eligible for this review. These articles were analyzed for terminologies used to indicate clinically meaningful change, analytic method employed for calculation and proposed MCID values.

## Results

Among 18 studies included in this review, 12 (67%) used the terminology MCID to indicate clinically meaningful changes after TKA [14–25] (Table 1). All these 12 studies used the terminology MCID alone except for one study [25] which used both MCID and MIC. Of the remaining 6 (33%) studies, two studies [13, 26] used clinically important difference (CID) alone, one study [12] used MCIC alone, one study [27] used MID alone, and one study [28] used both MIC and MID. Hence, although MCID was the most frequently used terminology to indicate clinically important change after TKA, other related terminologies are employed in about one third of the available studies.

**Table 1:** Terminologies used to refer to clinically meaningful change

Citation	Test	Terminology
Quintana et al., 2006 [16]	WOMAC and SF-36	MCID
Escobar et al., 2007 [14]	WOMAC and SF-36	MCID
Chesworth et al., 2008 [13]	WOMAC*	CID
Escobar et al., 2013 [17]	WOMAC*	MCID
Clement et al., 2014 [18]	OKS and SF-12 PCS	MCID
Escobar et al., 2014 [15]	WOMAC*	MCID
Keurentjes et al., 2014 [26]	SF-36	CID
Kiran, et al., 2014 [19]	OKS	MCID
Maratt et al., 2015 [12]	WOMAC*	MCIC
Kiran, et al., 2015 [27]	OKS	MID
Beard et al., 2015 [28]	OKS	MIC MID
Bin Abd Razak et al., 2016 [20]	OKS and SF-36 PCS	MCID
Lee et al., 2017 [21]	KSS	MCID
Berliner, et al., 2017 [22]	SF-12 PCS	MCID
Ingelsrud et al., 2018 [29]	OKS	MIC
Lizaur-Utrilla et al., 2019 [23]	KSS	MCID
Blevins et al., 2019 [24]	SF-12	MCID
Clement et al., 2019 [25]	SF-12	MCID MIC

*MCID* minimal clinically important difference, *MIC* minimal important change, *MID* minimal important difference, *CID* clinically important difference, *MCIC* minimal clinical important change, *TKA* total knee arthroplasty, *PROM* patient-reported outcome measures, *WOMAC* Western Ontario And McMaster University Arthritis Index, WOMAC* Western Ontario And McMaster University Arthritis Index reversed scoring system, *OKS* Oxford Knee Score, *KSS* Knee Society Score, *SF-36* 36-item Short Form survey, *SF-12* 12-item Short Form Survey

The methodology employed to calculate MCID of PROMs for TKA was variable in the included studies. Among 18 included studies, 12 (67%) studies [12–18, 21, 23, 25, 28, 29] employed anchor-based methods making them the most commonly used analytic methods to establish MCID. All of these 12 studies used only anchor-based method to determine MCID except for one study [23] which used both anchor-based and distribution-based methods. The remaining 6 (33%) studies [19, 20, 22, 24, 26, 27] used only distribution-based methods to calculate MCID.

Of the 12 studies employing anchor-based methods, receiver operator characteristic (ROC) curve analysis was the most frequently used method with as many as 7 (58%) studies employed it; either alone [12, 13] or in combination with mean change and/or regression analysis methods [17, 23, 25, 28, 29] (Table 2). The mean change method was the second most employed anchor-based method as it was used in a total of 6 (50%) studies; either alone [14–16] or along with other anchor-based methods [10, 28, 29]. The mean difference method was used in 3 (25%) studies along with other anchor-based methods to establish MCID [23, 25, 28]. Additionally, 2 (17%) studies [18, 21] utilized linear regression analysis to establish MCID and 1 (8%) study [29] employed logistic regression analysis.

**Table 2:** Methods used to calculate minimal clinically important difference

Citation	Test	Method	Anchor question	Transitional scale
Quintana et al., 2006 [16]	WOMAC and SF-36	Mean change	Not specified	5-point scale
Escobar et al., 2007 [14]	WOMAC and SF-36	Mean change	Not specified	5-point scale
Chesworth et al., 2008 [13]	WOMAC*	ROC	“Compared to when they went on the wait list for surgery, they were better, worse or the same”	15-point scale
Escobar et al., 2013 [17]	WOMAC*	Mean change ROC	“Compared to before surgery, how would you rate pain (functional limitation) in the same knee?” “Was surgery worthwhile?” “What is your global level of satisfaction with surgical management?”	5-point scale
Clement et al., 2014 [18]	OKS and SF-12 PCS	Linear regression analysis	“How well did the surgery relieve pain in your affected joint?” “How well did the surgery increase your ability to perform regular activities?”	5-point scale
Escobar et al., 2014 [15]	WOMAC*	Mean change	Not specified	5-point scale
Maratt et al., 2015 [12]	WOMAC*	ROC	“How much did your knee surgery improve the quality of your life?	6-point scale
Beard et al., 2015 [28]	OKS	Mean difference Mean change and ROC	“Overall, how are your problems now, compared to before your operation?”	5-point scale
Lee et al., 2017 [21]	KSS	Linear regression analysis	How would you rate the overall results of the surgery for your knee condition?”	6-point scale
Ingelsrud et al., 2018 [29]	OKS	Mean change Logistic regression and ROC	“How are your knee problems now compared to prior to operation?”	7-point scale
Lizaur-Utrilla et al., 2019 [23]	KSS	Mean difference ROC and 0.5 SD	“Compared to before surgery, how would you rate the pain in your knee and the ability to perform regular activities now?	5-point scale
Clement et al., 2019 [25]	SF-12	Mean difference ROC	“How much did the knee replacement surgery improve the quality of your life?”	5-point scale
Kiran et al., 2014 [19]	OKS	0.5 SD	Distribution-based method	–
Keurentjes et al. 2014 [26]	SF-36	0.8 SD	Distribution-based method	–
Kiran, et al., 2015 [27]	OKS	0.5 SD	Distribution-based method	–
Bin Abd Razak et al., 2016 [20]	OKS and SF-36 PCS	0.5 SD	Distribution-based method	–
Berliner, et al., 2017 [22]	SF-12 PCS	0.5 SD	Distribution-based method	–
Blevins et al., 2019 [24]	SF-12	0.5 SD	Distribution-based method	–

*MCID* minimal clinically important difference, *TKA* total knee arthroplasty, *PROM* patient-reported outcome measures, *WOMAC* Western Ontario And McMaster University Arthritis Index, WOMAC* Western Ontario and McMaster University Arthritis Index reversed scoring system, *OKS* Oxford Knee Score, *KSS* Knee Society Score, *SF-36* 36-item Short Form survey, *PCS* physical component summary, *SF-12* 12-item Short Form Survey, *ROC* receiver operator characteristics, *0.5 SD* half of a standard deviation, , *0.8 SD* four fifths of a standard deviation

Among the distribution-based methods, four studies [14, 16, 25, 28] reported MDC and one study [17] reported standard error of measurement (SEM) to assess the reliability of the MCID estimates calculated by the anchor-based approaches in those studies.

Apart from different analytical methods employed to calculate MCID, there was variation in the methodology among different studies even when they used the same analytical method. For instance, the studies employing ROC curve analysis to calculate MCID have used different transitional scales, cut-offs for area under the curve (AUC), and anchor questions (Table 2). Precisely, four studies used a 5-point transitional scale [17, 23, 25, 28], one study each employed 6-point [12], 7-point [29], and 15-point [13] scales in the methodology. Moreover, cut-off applied for AUC that defines the diagnostic ability of MCID calculated was inconsistent across various studies with wide range from 0.55 to 0.84. Additionally, a minimum AUC cut-off value of 0.7 is recommended to ensure optimal diagnostic reliability of this method and an increase in the AUC value indicates higher predictive accuracy [30]. However, one of the studies in this review calculated MCID of SF-12 with a reported AUC of less than 0.7 which questions the reliability of such an estimate [25]. Similarly, among seven studies that used a distribution-based method, six studies [19, 20, 22–24, 27] used 0.5 times the standard deviation (SD) to calculate MCID while one study [26] used 0.8 times the SD. Hence, there was variation in the methodology applied which using an individual analytical method used to calculate MCID among various studies.

The variation in methodology was also found among studies which calculated MCID in the context of individual PROMs (Table 3). For instance, there were 6 (33%) studies which reported the MCID of WOMAC employing either mean change [14, 17, 22], ROC [12, 13], or both [15]. Furthermore, these studies reported MCID values of WOMAC at varied time-points of analysis ranging from 6 months to 2 years. Similarly, among six studies reporting MCID values for OKS, three studies [18, 28, 29] used anchor-based methods while the other three studies [19, 20, 27] used distribution-based methods and evaluated MCID at varied time-points of follow-up ranging from 6 months to 5 years.

**Table 3:** Proposed minimal clinical important difference (MCID) values after total knee arthroplasty

Test	Citation	Follow-up	Subset	Method	Value	Variability (if reported)	Measurement error (if reported)
WOMAC	Quintana et al., 2006 [16]	6 months	Pain	Mean change	22.6		22.9
			Function		17.7		12.7
			Stiffness		12.9		29.1
	Escobar et al., 2007 [14]	2 years	Overall	Mean change	15.0		
			Pain		27.9		22.4
			Function		20.8		13.1
			Stiffness		21.4		29.1
	Chesworth et al., 2008 [13]	1 year	Pain	ROC	36.0		
			Function		33.0		
	Escobar et al., 2013 [17]	1 year	Pain	Global	30.0		8.6
				Mean change	29.9	27.1 to 32.6	
				ROC	20.5	20.2 to 20.9	
				Additional anchors	27.0		
			Function	Global	32.0		5.1
				Mean change	31.1	28.3 to 33.9	
				ROC	24.2	23.6 to 24.7	
				Additional anchors	20.0		
	Escobar et al., 2014 [15]	1 year	Pain	Mean change	29.0	27.0 to 31.1	
			Function		32.4	30.5 to 34.2	
	Maratt et al., 2015 [12]	2 years	Pain	ROC	31.3		
			Function		26.9		
			Stiffness		25.0		
OKS	Clement et al., 2014 [18]	1 year	Pain	Regression analysis	5.0	4.4 to 5.5	
			Function		4.3	3.8 to 4.8	
	Kiran et al., 2014 [19]	6 months	Overall	0.5 SD	4.9		
	Kiran et al., 2015 [27]	2 years	Overall	0.5 SD	4.7 at 1 year and 5.0 at 2 years		
	Beard et al., 2015 [28]	6 months	Overall	Mean change	9.0		4.0
				Mean difference	5.0		
				ROC	7.0		
	Bin Abd Razak et al., 2016 [20]	5 years	Overall	0.5 SD	5.0		
OKS	Ingelsrud et al., 2018 [29]	1 year	Overall	Mean change	10.0	8.0 to 11.0	
				ROC	9.0	6.0 to 15.0	
				Regression analysis	8.0	6.0 to 9.0	
KSS	Lee et al., 2017 [21]	2 years	Knee score	Regression analysis	5.9	3.9 to 7.8	
			Function score		6.4	4.4 to 8.4	
	Lizaur-Utrilla et al., 2019 [23]	2 years	Knee score	Mean difference	7.2	5.1 to 7.8	
				ROC	8.9		
				0.5 SD	7.2	5.3 to 9.0	
			Function score	Mean difference	9.7	7.3 to 10.2	
				ROC	10.3		
				0.5 SD	6.3	5.0 to 8.1	
SF-36	Quintana et al., 2006 [16]	6 months	Physical functioning	Mean change	10.4		20.2
			Role physical		7.8		28.7
			Bodily pain		12.8		42.3
			General health		0.1		27.0
			Vitality		5.4		29.7
			Social functioning		8.8		42.1
			Role emotional		2.4		28.1
			Mental health		0.8		23.9
	Escobar et al., 2007 [14]	2 years	Global	Mean change	10.0		
			Physical functioning		11.1	5.8 to 16.3	19.5
			Role physical		13.2	3.5 to 22.8	26.9
			Bodily pain		6.7	− 0.4 to 13.8	37.9
			General health		− 7.3	− 11.3 to − 3.3	27.4
			Vitality		3.4	− 2.2 to 9.1	41.2
			Social functioning		6.2	− 1.7 to 14.0	28.5
			Role emotional		2.4	− 9.2 to 14.1	29.8
			Mental health		4.0	− 1.7 to 9.7	24.2
	Keurentjes et al., 2014 [26]	1.5 to 6 years	Physical functioning	0.8 SD	16.7	15.5 to 18	
			Role physical		33.4	31.2 to 36	
			Bodily pain		16.2	15.1 to 17.5	
			General health		15.7	14.7 to 16.9	
			Vitality		16.7	15.6 to 18.0	
			Social functioning		19.9	18.6 to 21.5	
			Role emotional		33.3	31.3 to 36.2	
			Mental health		14.1	13.2 to 15.2	
	Bin Abd Razak et al., 2016 [20]	5 years	PCS	0.5 SD	10.0		
SF-12	Clement et al., 2014 [18]	1 year	PCS	Regression analysis	4.5 (Pain relief)	3.9 to 5.2	
					4.8 (Function)	4.2 to 5.4	
	Berliner, et al., 2017 [22]	1 year	PCS	0.5 SD	5.0		
	Blevins et al., 2019 [24]	2 years	PCS	0.5 SD	5.0		
			MCS		5.4		
	Clement et al., 2019 [25]	1 year	PCS	Mean difference	1.8		
				ROC	2.7		
			MCS	Mean difference	1.5		
				ROC	−1.4		
	Clement et al., 2014 [18]	1 year	PCS	Regression analysis	4.5 (Pain relief)	3.9 to 5.2	
	Berliner, et al., 2017 [22]				4.8 (Function)	4.2 to 5.4	
		1 year	PCS	0.5 SD	5.0		
	Blevins et al., 2019 [24]	2 years	PCS	0.5 SD	5.0		
			MCS		5.4		
	Clement et al., 2014 [18]	1 year	PCS	Regression analysis	4.5 (Pain relief)	3.9 to 5.2	
	Berliner, et al., 2017 [22]	1 year	PCS	0.5 SD	4.8 (Function)	4.2 to 5.4	

*MCID* minimal clinically important difference, *TKA* total knee arthroplasty, *PROM* patient-reported outcome measures, *WOMAC* Western Ontario and McMaster University Arthritis Index, WOMAC* Western Ontario and McMaster University Arthritis Index reversed scoring system, *OKS* Oxford Knee Score, *KSS* Knee Society Score, *SF-36* 36-item Short Form survey, *SF-12* 12-item Short Form Survey, *ROC* receiver operator characteristics, *0.5 SD* half of a standard deviation, *0.8 SD* four fifths of a standard deviation

The calculated MCID values of PROMs for TKA also showed wide variation (Table 3). MCID values reported for WOMAC sub-scales in the included studies were 20.5 to 36.0 for pain, 17.6 to 33.0 for function and 12.9 to 25.0 for stiffness [12–17]. MCID values of OKS reported by studies were 4.7 to 10 points [18–20, 27–29]. Even although these MCID values for OKS show variation, it was less apparent than the wider variation of MCID values reported for WOMAC. Additionally, MCID for OKS reported by distribution-based approaches were lower (4.7 to 5 points) compared to those calculated by anchor-based approaches (5 to 10 points). Among anchor-based methods, MCID reported by mean change was noted to be higher (9 to 10 points) compared to other methods (5 to 9 points) [19, 27]. Regarding the original KSS, two studies reported MCID values at 2 years’ follow-up as 1.9 to 9.0 for the knee score (KSS-KS) component and 4.4 to 10.2 for the function score (KSS-FS) component [20, 31]. There were no studies that reported MCID values for the new KSS scoring system. Four studies reported MCID for SF-36 score at 6 months to 5 years after TKA [14, 16, 20, 26]. Two [14, 16] of these studies utilized anchor-based methods to report the MCID of the Spanish translated and validated version of SF-36. Other two studies utilized distribution-based methods to report the MCID of the original scoring system of SF-36 and its Dutch translated system [20, 26] (Table 3). Regarding SF-12, four studies [18, 22, 24, 25] reported MCID at 1 or 2 years after TKA using anchor- and distribution-based methods. Two [18, 22] of these studies reported MCID only for the physical component summary component of SF-12 and two other studies reported MCID for both mental and physical components [24, 25]. MCID values reported for the physical component summary was in the range 1.8 to 5 points and that for the mental component was − 1.4 to 5.4 points.

## Discussion

The orthopedic surgeons should have a detailed knowledge of the MCID of commonly used PROMs for patients undergoing TKA in terms of variations in the used terminology, optimum methodology of MCID calculation, and a critical overview of the available MCID values. One of the key findings of this review was that MCID was the most common terminology used to indicate clinically important change in PROMs. However, other similar and confusing terminologies are mentioned in about one third of the available studies on the topic. The analytical methods used to calculate MCID and its available values for clinical use are highly variable. Nonetheless, the anchor-based methods, especially ROC-curve analysis, are most commonly used to calculate MCID in conjunction with other methods. MCID values that may be routinely used with caution are 20.5 to 36.0, 17.6 to 33.0 and 12.9 to 25.0 for pain, function and stiffness sub-scales of WOMAC score, respectively, and 4.7 to 10 for OKS.

### MCID and related terminologies

Jaeschke et al. [32] introduced the concept of MCID in 1989 as the smallest difference in scores in the domain of interest which patients perceive as beneficial and which would mandate, in the absence of troublesome side effects and excessive cost, a change in the patient’s management. Subsequently, the term MCID has been generically used to indicate clinically meaningful change in a wide variety of clinical contexts, irrespective of the analytic method used in its estimation. Our study found that the terminology “MCID” has been used in two thirds of the studies that calculated the estimate of clinically meaningful change in PROMs after TKA. However, several other related terminologies like MCIC, MIC, MID, CID, and MDC may be confused with MCID and need further consideration. This will help physicians to improve their understanding of the distinctions between various terminologies and reach consensus regarding their appropriate utility in clinical and research setting.

Minimal clinically important change (MCIC) is a distinct terminology but with a similar definition as MCID and is utilized synonymously with it in a study to predict the rates of satisfaction after TKA [12]. Another terminology used in studies [18, 28, 29] related to TKA is called MIC which is defined as the change in the health status in a single group or single individual over a period of time. The MIC is specifically utilized to indicate changes “within a group” (calculated using mean change method) or more importantly “within an individual” (calculated using ROC-curve analysis). As the group averages fail to capture the changes “within an individual level”, the use of MIC that is calculated by ROC analysis is of great relevance to assess the progress in the individual patients in clinical practice. Hence, we recommend the specific use of MIC to indicate changes in the health status “within an individual”, albeit the use of MCID to non-specifically indicate the changes in health status across all clinical contexts.

Minimal important difference (MID), defined as the differences in the health gain or loss between two independent groups of patients, is applicable in setting of clinical trials [28]. The MID is calculated by differences in the mean scores in patients reporting themselves as “little better” and “about the same”. Accordingly, two studies have used this terminology while calculating the estimate of minimum important clinical change in PROMs after TKA [27, 28]. However, such similar contextual application of MID was not supported in other subsequently published studies which used MCID to compare health status between the comparison groups [23, 25]. Nonetheless, the concept of MID may be more relevant for clinical research where this terminology may be used interchangeably with MCID [33].

The concept of MID represented by above terminologies has been criticized in the context of TKA as one would expect larger than minimum improvements after TKA to be clinically more relevant [13, 26]. Accordingly, the terminology clinically important difference (CID) was used in two studies to indicate clinically relevant changes after TKA that are not necessarily minimum [13, 26]. CID was defined as the difference in scores of an outcome measure that is perceived by patients as beneficial or harmful. It is calculated using a transitional group that reports more than minimal improvements such as “good deal better” in contrast with MCID which involves the use of a transitional group showing slight or minimal improvements after TKA that is the “somewhat better” group [13]. Future studies should evaluate the relative clinical relevance between CID and MCID to improve our understanding for their application in clinical practice and research related to TKA.

In contrast to all the above terminologies indicating clinical significance, minimum detectable change (MDC) is a purely statistical concept. MDC is defined as the minimal change that can be detected taking the measurement error into account [31]. The concept of MDC is based on the standard error of measurement (SEM) and is used in reliability assessment of the calculated MCID values. For instance, MCID that is less than MDC is questioned for its reliability as it lies within the bounds of measurement error of the PROM. Conversely, with a MCID greater than MDC_95,_ it is possible to state with 95% of confidence that the change in scores is outside the bounds of measurement error and thus reflecting a true change [16]. Hence MDC acts as a reasonable starting point to detect the reliability of the calculated MCID values but cannot reflect clinically important change in a PROM.

With this background on the nuances in the MCID-related terminologies, it is recommended to maintain a standardized terminology in the literature in order to avoid the confusion among the clinicians and researchers. Considering the continued and increasing utilization of the term MCID since its inception in 1989, it seems that the term MCID has stood the test of time and should be the choice of terminology used in clinical practice and research in future [23, 25]. Nonetheless, the specific use of MIC for indicating changes within an individual is potentially advantageous considering its relevance in clinical practice.

### Analytical methods for calculating MCID

There is a wide variation in the analytic methods used to calculate MCID that is presented in this review. Nine distinct methods that are currently employed to calculate MCID can be categorized into anchor-based and distribution-based methods [31].

Anchor-based methods use an independent tangible criterion in the form of a clinical or patient-based anchor question to calculate MCID. The responses to these anchor questions are typically used to assign the population under study into transitional groups. For instance, the response to an anchor question “Compared to before surgery, how would you rate pain in the same knee?” is used to establish transitional groups such as “great deal better,” “somewhat better,” “equal,” “somewhat worse” and “great deal worse” on a typical 5-point global rating of change (GRC) scale [17]. Thereafter, the baseline pre-TKA and post-TKA PROM scores are analyzed in four distinct methods (mean change, mean difference, ROC, and regression analysis) emphasizing on the transitional group that reports minimal change (“somewhat better”). Two third of the studies included in this review employed anchor-based methods; making them the most-used analytic methods to establish MCID of PROMs for patients treated with TKA (Table 2).

Among anchor-based methods, ROC-curve analysis was the most-used method to calculate MCID. It entails establishing the threshold of MCID by a single point on the ROC curve that has maximum sensitivity and specificity to dichotomize the patients into those who achieved the clinically meaningful change (somewhat better and great deal better) and to those who did not (equal, somewhat worse and great deal worse) [34]. The United States Food and Drug Administration recommends ROC-curve analysis as the best available method to establish MCID for “within an individual” analysis [35]. However, MCID determined by ROC-curve analysis may not be ideal for analyzing changes “within or between the groups as it involves a single point estimate on ROC curve with no confidence intervals which is equivalent to pointing at a single individual out of the whole group. Furthermore, the results of this review reflect that the heterogeneities in the methodology of the studies which calculated MCID related to TKA in terms of different transitional scales, ROC-curve cut-offs for AUC, and anchor questions used (Table 2).

The mean change method was the second most employed anchor-based method to calculate MCID among the studies included in this review. MCID is calculated using the mean change method by estimating the absolute change in the mean PROM scores from baseline to follow-up in the sub-group of the patients who report themselves as “somewhat better” [32]. As this method entails the study of longitudinal changes in one group over a period of time, it is best used for cohort studies. Additionally, at an individual-level application it is deemed to misclassify certain individuals as not having a change when the magnitude of their change falls below the group mean. The mean change method was the third most-employed anchor-based method among the studies included in this review. In contrast to the mean change method, the mean difference method calculates MCID by estimating the difference in PROM score between two transitional groups (like “somewhat better” and “no change”), making it more relevant in clinical trials while comparing intervention and control groups. Regression analysis is the fourth most-employed anchor-based approach that uses linear or logistic regression modeling to the mean score differences (from baseline to follow-up) to establish MCID. In the simplest form of linear regression, the slope of the linear relation between the differences in the PROM scores (independent variables) and transitional responses (dependent variable) is used to establish MCID [18, 21]. In logistic regression analysis, the non-linear relationship between the transitional responses (dependent variables) and all the confounding factors that can possibly affect it such as age, sex and baseline PROM scores (independent variables) are analyzed to establish MCID [29]. It has been proposed as one of the least biased methods for establishing the MCID for “between the groups” analysis that involves comparison of groups with different parametric characteristics with independent confounding influences on MCID [29].

Apart from the drawbacks of the individual methods highlighted above, there are few shortcomings of anchor-based methods that warrant caution. Firstly, the anchor questions used to establish MCID are not validated, in addition, to the heterogeneity in the methodology pointed earlier (Table 2). As the calculation of MCID is dependent on both these factors, they may be responsible for wide variability in MCID values (Table 3). Secondly, using a single anchor question to calculate MCID has been a cause of concern as it is difficult to completely capture the changes following TKA with one anchor question. Thirdly, anchors have been criticized for their susceptibility to recall bias (the patient’s memories of the prior health state may often be inaccurate) and the tendency to be affected by the patient’s current status. The above-mentioned limitations in the anchor-based methods used to calculate the available MCID values of PROMs related to TKA warrants caution during their interpretation and clinical application.

The distribution-based methods, in contrast to anchor-based methods, are grounded on the statistical significance with no direct relationship to clinical significance. While standard deviation (SD) is the most frequently employed statistical method to determine MCID, standard error of measurement (SEM) and MDC report the measurement error used to assess the reliability of the MCID calculated by anchor-based approaches. The rationale for using SD is based on an assumption that half of the SD of the pre-treatment scores most likely approximates to a moderate effect size [30]. Seven out of 18 studies in this review employed distribution-based methods to calculate MCID of PROMs related to TKA (Table 2). 

In contrast to SD, which is sample-dependent, standard error of measurement (SEM) and MDC denote the measurement error in the PROM instrument, independent of the patient population. Although described as one of the methods used to establish MCID, we believe that SEM and MDC are statistical entities that best denote the measurement error with no consistent relationship with MCID and therefore cannot independently replace it.

The use of distribution-based methods to establish MCID has been challenged for not providing direct information regarding a patient’s perspectives of change. As they are more statistical than clinical it is believed that they do not address the “clinical” part of “minimal clinical important difference” [11]. Secondly, although the magnitude of change determined by SD or effect size is certainly statistically significant, it might not necessarily be the reliable cut-off to establish MCID. Thirdly, as SD is sample dependent, MCID obtained by using SD cannot be generalized to other populations. Due to such inherent limitations, distribution-based methods are not employed alone and rather are used as a supplement to the anchor-based methods in the determination of MCID .

Considering multiple analytical methods with heterogeneity in methodology across studies and inherent limitations, multiple MCID values with wide variations have been reported for the same PROM (Table 3). Moreover, there is no established consensus yet on the best available approach to calculate MCID. Although it has been traditionally recommended to synthesize a smaller range of values by incorporating anchor- and distribution-based methods together, conceptually referred to as triangulation; it is interesting to note the paucity of such attempts in the literature pertaining to TKA [36]. Although a modified Delphi model has been proposed to obtain a reasonable consensus in other specialties, it is important to recognize that these judgments cannot be objectively verified. Nonetheless, researchers are recommended to employ validated standardized methodology with multiple anchor questions and triangulation to completely capture the changes after TKA along with consistent reporting of the measurement error to ensure the reliability of calculated estimates.

### Available MCID values of commonly used PROMs related to TKA

The commonly used PROMs evaluating the clinical outcomes of TKA use either disease- or joint-specific PROMs such as WOMAC, OKS and KSS or generic PROMs such as SF-36 and SF-12 which evaluate health-related quality of life.

Western Ontario and McMaster Universities Arthritis Index (WOMAC) is a validated, 24-item, disease-specific questionnaire used to evaluate patients with hip or knee OA, with three sub-scales measuring pain (five items), stiffness (two items), and function (17 items) [7]. Each of the items has five possible responses with scores of 0 to 4 for each response with a maximum score of 96. Six out of 18 studies reported MCID values of WOMAC score using different analytical methods and at varied time-points of analysis ranging from 6 months to 2 years (Table 3). These studies reported a wide range of MCID values for WOMAC sub-scales ranging from 20.5 to 36.0 for pain, 17.6 to 33.0 for function and 12.9 to 25.0 for stiffness. The possible reasons for the wide variability in MCID values are inconsistencies in the analytic methods as highlighted previously.

Oxford Knee Score (OKS) is a validated, knee-joint-specific, 12-item questionnaire with five items assessing pain and seven items for function. Each item has equal weightage (0 to 4) with a possible score ranging from 0 to 48 and a higher score indicating better outcomes [6]. Six studies reported MCID values of OKS employing different methods using different analytical methods at varied time-points of follow-up ranging from 6 months to 5 years (Table 3). Overall, MCID values of OKS demonstrated better convergence compared to WOMAC ranging from 4.7–10.0 points.

The original KSS proposed in 1989 is a knee-joint-specific questionnaire with two sub-scales, knee rating (KSS-KS, 0–100 points) and function score (KSS-FS, 0–100 points) [3]. The KSS-KS is further categorized into pain (0–50 points) which is patient-reported and knee score (0–50 points) that is clinician rated in terms of range of motion (ROM), alignment and stability. Two studies reported MCID value of original KSS sub-scales at 2 year follow-up using different analytical methods (Table 3). As a clinician-completed scoring system, concerns have been raised regarding its validity which has led to the proposition of new KSS [37]. However, none of the studies in this review have reported MCID values of new KSS.

SF-36 is a generic instrument used to assess health-related quality of life with eight domains and two summary scales: physical component summary and mental component summary [4]. Four studies reported the MCID of SF-36 at 6 months to 5 years after TKA using different analytical methods (Table 3). The 12-item Short Form survey (SF-12) is a consolidated version of SF-36 with 12 items and eight scales or domains [5]. Four studies reported a MCID of SF-12 at 1 to 2 years after TKA using anchor- and distribution-based methods (Table 3). The reported MCID values for SF-12 physical component summary ranges from 1.8 to 5.0 points, it is between − 1.4 to 5.4 points for the mental component summary.

The limitations in methodology of MCID calculation warrants caution to clinicians before these MCID values in clinical practice.

### General considerations while using MCID values

The concept of MCID is associated with certain inherent limitations that the clinicians and researchers need to be mindful of before clinical application. Firstly, MCID is a context-specific entity. A context not only includes the type of disease (osteoarthritis) or treatment (TKA) but also comprises of population characteristics like age, sex, socio-economic factors, baseline disease severities and patient expectations. Hence, MCID is not a fixed attribute and should be cautiously applied across varied patient populations. Secondly, MCID is specifically meant to capture an individual’s response to treatment rather than the mean experience of the entire group. MCID reported as a single point estimate using group mean scores runs the risk of misclassifying certain individuals as non-responders when their improvement falls below the group mean. Therefore, MCID derived from group mean scores should be judiciously applied in clinical practice to detect individual-level changes. Instead, MCID by ROC analysis for individual-level application serves a better purpose in this regard [35]. Thirdly, MCID is commonly used for determining sample sizes in clinical research based on an assumption that it ensures clinical significance to the statistically established significance. Such assumptions have been lately questioned and caution is advised while application of MCID in power analysis [31]. Due to the aforementioned limitations in its use, established MCID values need to be utilized judiciously, considering the specific context of application.

### Future directions

The concept of MCID has been evolving over the past few years with many areas of ongoing research. In our opinion, progress in the following areas will permit the better use of this alluring concept. Firstly, consensus on the appropriate use of relevant terminologies and standardizing the methods of calculation are much needed to go beyond the existing state of conflict and to use MCID as a powerful outcome metric. Additionally, large organizations and consortia of researchers can provide consensus-based periodic updates on MCID values of commonly used PROMs [11]. Secondly, to address the variability of MCID values, the preferred approach is to synthesize a smaller range of values by incorporating anchor- and distribution-based methods together using triangulation as they complement each other. Thirdly, as MCID is a context-specific entity, the best way to obtain reliable estimates is to establish MCID that is specific to the clinical settings using standard patient populations and validated linguistic questionnaires, something that is feasible at large-volume centers. With progress in the aforementioned areas, the utility of MCID can be vastly improved that can make it a powerful tool in clinical research besides aiding in clinical decision-making and better treatment practices.

## Conclusion

Of the existing terminologies, MCID remains the most-used and anchor-based methods are the most-used analytic methods to calculate it. The MCID of WOMAC and OKS are reported in most of the studies with estimates ranging from 20.5 to 36.0 for pain, 17.6 to 33 for function, and 12.9 to 25 for stiffness sub-scales of WOMAC score and 4.7 to 10 points for OKS. As it is a context-specific value, the judicious use of published MCID values is advisable both in clinical and research settings. Although, there is no ideal method, synthesizing a smaller range in MCID estimates by triangulation of both anchor- and distribution-based approaches is recommended. However, due to the paucity of such attempts in the literature pertaining to TKA, MCID determined by ROC is regarded as most suitable for “within an individual” analysis in clinical practice and estimates obtained by regression analysis are considered least biased for “between the groups” analysis in clinical research comparing study and control groups.

## Data Availability

The data presented in this manuscript is available on the PubMed and EMBASE databases.

## Abbreviations

CID: Clinically important difference
MCIC: Minimal clinically important change
MCID: Minimal clinically important difference
MDC: Minimum detectable change
MIC: Minimal important change
MID: Minimal important difference
KSS: Knee Society Score
OKS: Oxford Knee Score
PROMs: Patient-reported outcome measures
SF-36: 36-item Short Form survey
SF-12: 12-item Short Form survey
TKA: Total knee arthroplasty
WOMAC: Western Ontario and McMaster Universities Arthritis
